# Optimization of Cutting Parameters for Deep Hole Boring of Ti-6Al-4V Deep Bottle Hole

**DOI:** 10.3390/ma16155286

**Published:** 2023-07-27

**Authors:** Wanzhong Li, Huan Zheng, Yazhou Feng

**Affiliations:** Mechanical Engineering College, Xi’an Shiyou University, Xi’an 710065, China; liwanzhong@xsyu.edu.cn (W.L.); zhenghuan21@outlook.com (H.Z.)

**Keywords:** deep hole boring, deep bottle hole, response surface method, regression analysis, parameter optimization

## Abstract

In this study, the cutting parameters for machining deep bottle holes (deep holes with complex profiles and length-to-diameter ratio greater than 10) were optimized based on cutting simulation, a regression analysis genetic algorithm, and experimental validation. The influence of cutting parameters on cutting force and cutting temperature was analyzed using the response surface method (RSM), and the regression prediction model of cutting parameters with cutting force and most cutting temperature was established. Based on this model, multi-objective optimization of cutting force *F_x_* and material removal rate *Q* was carried out based on a genetic algorithm, and a set of optimal cutting parameters (*v* = 139.41 m/min, *a_p_* = 1.12 mm, *f* = 0.27 mm/rev) with low cutting force and high material removal rate were obtained. Finally, based on the optimal cutting parameters, the machining of TC4 deep bottle holes with a length-to-diameter (L/D) ratio of 36.36 and a roughness of Ra 3.2 µm was accomplished through a deep hole boring experiment, which verified the feasibility of the selected cutting parameters and provided a certain reference for the machining of this type of parts.

## 1. Introduction

**Figure 1:**
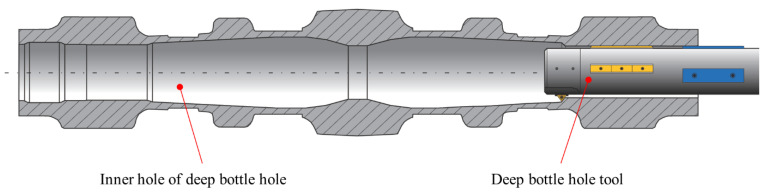
Deep bottle hole.

Ti-6Al-4V (TC4) is an α + β phase titanium alloy that is widely used in aerospace, automotive manufacturing, and biomedical devices due to its excellent corrosion resistance and high strength [1,2]. Deep bottle hole parts such as aircraft landing gear made of TC4 are usually machined based on special deep bottle hole tools or special electrolytic equipment whose profiles exist for weight reduction and efficiency [3,4]. Their inner hole characteristics are shown in Figure 1. The presence of such holes poses a great challenge for deep hole machining. However, since special electrolytic equipment requires the design of different tool electrodes for the machining of holes with different internal profiles, its machining applicability is low, so special deep bottle hole tools are usually used to machine the deep bottle holes. Due to the low thermal conductivity, high friction coefficient, low modulus of elasticity, and high affinity of elements of TC4 [5,6,7], this material is often faced with machining challenges such as high cutting forces and high cutting temperatures in deep hole boring [8].

To obtain a good surface quality of deep bottle holes and high machining efficiency, reasonable cutting parameters need to be selected, which include cutting speed (*v*), depth of cut (*a_p_*), and feed rate (*f*) [9]. The reduction in cutting forces, cutting temperatures, and improvement of hole surface quality can be achieved by optimizing the abovementioned cutting parameters [10,11]. Abdelhafeez et al. [12] conducted a drilling test on CR4 steel using double flute twist drills of different diameters to study the effect of feed rate and tool diameter on tool rear face wear and inner hole quality. It was found that the tool’s rear face wear increased when the feed rate was reduced and a larger diameter tool was used. Al-Tameemi et al. [13] investigated the effect of cutting parameters (spindle speed and feed rate) and three types of tool coatings (TiN/TiAlN, TiAlN, and TiN) on the surface finish, shape, and dimensional tolerances of drilled holes. It was found that all holes in this study were oversized, regardless of the tool coating or cutting parameters used. Aresh et al. [14] studied the effect of tool geometry parameters such as rake angle and cutting parameters such as depth of cut on cutting forces to explain the stress state at the cutting edge, and the results show that a cutting tool with a 20° rake angle achieves efficient cutting. Feng et al. [15] carried out the design of deep hole trepanning tool and performed deep hole trepanning tests for TC10 titanium alloy at different cutting speed and feed rate, and the results show that chip morphology, tool wear, and hole surface quality can meet the machining requirements when the feed rate is in the range of 0.10–0.15 mm/r and the cutting speed is less than 27 m/min, and the machining of TC10 deep hole tube was realized with an L/D ratio of 21.

The finite element cutting simulation can input the cutting parameters and set the boundary conditions to approximate the actual cutting process, which can not only output some cutting force, cutting temperature, energy, and other target parameters that are impossible or difficult to output by conventional testing means but also optimize the cutting parameters and predict the machining results through the results of cutting simulation to achieve the purpose of cost saving. The magnitude of cutting force and cutting temperature affects the whole cutting process and needs to be controlled reasonably. When the cutting force and cutting temperature are well controlled, not only can the surface quality of the workpiece be improved, but also the tool wear can be reduced. Jiang et al. [16] established a TC4 three-dimensional finite element model to analyze the cutting process with different tool parameters and found that residual tensile stresses are generated on the inner cutting surface and residual compressive stresses on the outer cutting surface when the cutting speed is 140 m/min. Guo et al. [17] established a meshless 3D milling simulation model of TC4 titanium alloy by using the smooth particle hydrodynamics (SPH) method to simulate the cutting forces with different cutting parameters and found that the cutting force is the most sensitive to the axial depth of cut, followed by the radial depth of cut, and it is least sensitive to the feed per tooth value. Liu et al. [18] established a finite element model (FEM) for numerical prediction of TC4 turning and analyzed the effect of LN2 low-temperature machining on residual stress distribution, and the results showed that LN2 cryogenic machining can effectively reduce residual tensile stresses, and the study provided some reference for future numerical prediction and suppression of residual stresses. Hu et al. [19] investigated the kinematics and material removal mechanisms of longitudinal bending hybrid ultrasonic vibration-assisted milling (LBVAM) through analytical studies, 3D finite element simulations, and corresponding experiments. Simulation and experimental results showed that the use of LBVAM can reduce surface roughness by 46.7% and cutting forces by 43.2% compared to conventional milling. Coelho et al. [20] performed experimental and numerical tests on deep drilling of Ti-6Al-4V ELI titanium alloy; analyzed cutting forces, geometric tolerances, and temperatures; and found that the use of high feed rate in drilling Ti-6Al-4V ELI can improve productivity and meet the needs of the biomaterials field within certain tolerances. However, cutting simulations and tests have focused on conventional turning and milling, and less research has been carried out on deep hole boring and even less on machining of deep bottle holes. At the same time, for the machining of deep bottle holes, the conventional deep hole machining method can only accomplish the machining of some deep bottle holes with a small depth of cut (<5 mm) and relatively small L/D ratio (<30).

In this study, firstly, we used the combination of the finite element method and the response surface method to analyze the effect of cutting parameters such as cutting speed, depth of cut, and feed rate on the cutting force and cutting temperature in each direction of deep hole boring TC4. Secondly, we established the regression model of three factors with cutting force and cutting temperature and optimized the cutting parameters. Finally, a deep bottle hole with a depth of cut of 5.5mm and an L/D ratio of 36.36 was machined.

## 2. Cutting Simulation

### 2.1. Cutting Simulation Solutions

As the cutting conditions involved in deep bottle hole tool are more complex if the cutting simulation is carried out directly on the tool as a whole, the calculation cost is incalculable, so the cutting simulation involved in this study mainly simulates the action process of the deep bottle hole tool insert part and workpiece. As there is less reference basis for cutting parameters when deep hole boring machining titanium alloy, the cutting parameters of deep hole boring of TC4 were selected according to the enterprise production machining experience, the range of cutting speed (*v*) is 50~140 m/min, the range of depth of cut (*a_p_*) is 1~3 mm, and the range of feed rate (*f*) is 0.2~0.4 mm/rev. To obtain the cutting force model in *x*, *y*, and *z* directions, the *v*, *a_p_*, and *f* were selected as the influencing factors, and three levels were set for each factor, as shown in Table 1.

**Figure 2:**
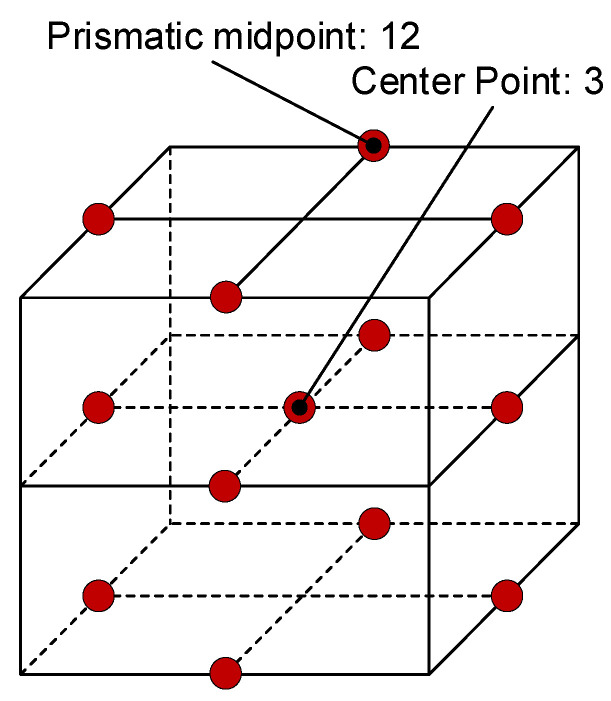
Box–Behnken model.

The simulation is based on the Box–Behnken response surface method to analyze the cutting force in different directions for three factors, and the design arrangement of this method is to take each test point at the prismatic midpoint of the square, which can effectively reduce the number of simulations. The Box–Behnken model is shown in Figure 2. The simulation was carried out at the midpoint based on 12 prismatic midpoints, and the number of center points was set to 3. A total of 15 sets of simulation tests were recorded.

### 2.2. Cutting Simulation Model

(1) Material constitutive model

In this study, the difficult-to-machine material TC4 was used as the cutting simulation and test machining material. During the cutting simulation, the material properties need to be set. The materials used in this cutting simulation were TC4 (workpiece) and YG8 cemented carbide (tool), and their material parameters are shown in Table 2.

The cutting process is a complex thermodynamically coupled process, and to be able to simulate the cutting process similar to the actual cutting state, an accurate material constitutive model must be established. Since the Johnson–Cook model integrates relevant parameters such as strain, strain rate, and temperature and can still express the thermoplasticity of the material under the conditions of high temperature rise and large strain rate and has good stability as well as strong material adaptability, this model was chosen as the material constitutive model with the following expressions

(1) $$\sigma =\left[A+B\varepsilon^{n}\right]\left[1+C\ln\left(\frac{\varepsilon }{\varepsilon_{o}}\right)\right]\left[1-{\left(\frac{\left(T-T_{r}\right)}{\left(T_{m}-T_{r}\right)}\right)}^{m}\right]$$

where σ is the equivalent stress; *A*, *B*, *C*, *m*, and *n* are material constitutive parameters; ε is the equivalent plastic strain; $\varepsilon_{o}$ is the reference strain rate; *T* is the material temperature; *T_m_* is the material melting point; and *T_r_* is the room temperature.

The plastic and damage parameters of the Johnson–Cook constitutive model for TC4 material are shown in Table 3.

In the metal cutting simulation, chip separation from the workpiece also occurs, and to describe this process more accurately, the corresponding material failure model needs to be introduced

(2) $$\omega =\sum \frac{\Delta {\bar{\varepsilon }}^{\;pl}}{{\bar{\varepsilon }}_{f}^{\;pl}}$$

where ω is the failure parameter; $\Delta {\bar{\varepsilon }}^{\;pl}$ is the equivalent plastic strain increment; and ${\bar{\varepsilon }}_{f}^{\;pl}$ is the failure strain.

When ω is greater than 1, the workpiece material fails, causing chip separation.

(2) Finite element model

**Figure 3:**
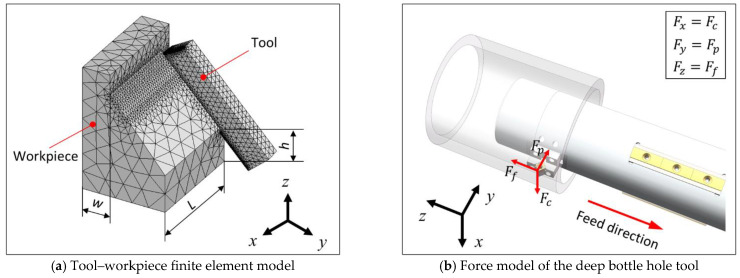
Tool–workpiece model.

To improve the simulation efficiency, the local area of the TC4 tube was taken for simulation. Figure 3a shows the finite element model of the local cutting area established in AdvantEdge, and since the left side of the workpiece is not involved in cutting, mesh refinement was carried out for the area on the right side of the workpiece that is actually in contact with the tool. In this tool–workpiece finite element model, the workpiece material is TC4, and its length *L*, width *w*, and height *h* are 5 mm, 1.5 mm, and 2 mm respectively. The material of the tool is YG8 cemented carbide with certain impact performance and no affinity with Ti elements, and the rake angle $\gamma_{0}$, relief angle $\alpha_{0}$, edge inclination angle $\lambda_{s}$, and main deflection angle $\kappa_{r}$ are 7°, 7°, 0°, and 50°. The tip radius $R_{n}$ was set to 0.04 mm, and the insert model was CCMT060204. Figure 3b shows the force model of the deep bottle hole tool, in which the tangential force *F_c_*, back force *F_p_*, and feeding force *F_f_* correspond to *F_x_*, *F_y_*, and *F_z_* in Figure 3a, respectively.

### 2.3. Analysis of Cutting Simulation Results

In this cutting simulation, the *v*, *a_p_*, and *f* are used as factors, and the cutting forces (*F_x_*, *F_y_*, and *F_z_*) in three directions and the cutting temperature (*T_n_*) are used as response values for cutting numerical simulation. The 15 sets of cutting simulation results based on Table 1 are shown in Table 4, where the values of *F_x_*, *F_y_*, and *F_z_* are the average values of the cutting forces in each direction in the stable cutting stage, and the value of *T_n_* is the average value of the maximum cutting temperature in the stable cutting stage.

The selection of the predictive model needs to be decided based on the *p*-value; the smaller the *p*-value, the higher the proven accuracy of the selected model. Since the quadratic regression model has the smallest *p*-value, it was chosen for regression analysis. In this paper, a quadratic regression model (3) is used to describe the effects of three factors on cutting force and cutting temperature, and the quadratic regression prediction model of each factor and cutting force and cutting temperature in each direction is established as follows

(3) $$Y=b_{0}+\sum_{i=0}^{k}b_{i}x_{i}+\sum_{i\leq j}^{k}b_{ij}x_{i}x_{j}+\sum_{i=0}^{k}+b_{ii}x_{i}^{2}$$

where Y is the estimated value of cutting force in each direction, b is the model coefficient, and x is the cutting parameter level.

The coefficient matrix can be expressed as follows

(4) $$\beta ={\left(X^{T}X\right)}^{-1}X^{T}X$$

where X is the matrix of simulation factors; β is the result matrix of the simulation.

The multiple regression models between the cutting forces in each direction, the cutting temperature, and the cutting parameters were established by converting the simulation test parameters and results into matrix form and combining them with the least squares regression method. Finally, based on the regression analysis of the numerical simulation results in Table 5, the obtained quadratic regression prediction models for *F_x_*, *F_y_*, *F_z_*, and *T_n_* are:

(5) $$F_{x}=100.9-0.1v-5.9a_{p}-196.39f-0.39va_{p}-0.22vf+4280a_{p}f$$

(6) $$\begin{array}{c} F_{y}=736.34-3.11v-224.28a_{p}-2944.86f-0.54va_{p}+8.72vf\\ +1635a_{p}f+0.008v^{2}+51.38a_{p}^{2}+2912.5f^{2} \end{array}$$

(7) $$\begin{array}{c} F_{z}=638.43-0.66v-171.36a_{p}-3425.7f-0.78va_{p}+3.39vf\\ +1890a_{p}f+0.004v^{2}+33.38a_{p}^{2}+3362.5f^{2} \end{array}$$

(8) $$\begin{array}{c} T_{n}=629.26+1.85v-34.72a_{p}-105.14f+0.19va_{p}-0.22vf\\ +72.5a_{p}f \end{array}$$

It should be noted that since the cutting force model and the cutting temperature model can change depending on the material, the cutting force model, and the cutting temperature model developed in this study are only applicable to TC4 material and are consistent with the above method when models for other materials need to be modeled.

To verify the validity of the regression prediction model, parameter evaluation and ANOVA analysis of Equations (5)–(8) are also required, and the main results are shown in Table 5. In this parameter evaluation, the significance level is 0.05, and the *p*-values of the four regression prediction models after the F-test are less than 0.05, indicating that the models are significant and can predict the regression values. The lack of fit is an important basis for assessing the reliability of the model, and the lack of fit of the four regression prediction models is all greater than 0.05, indicating that the lack of fit is not significant, which further verifies the reliability of the model. The *R^2^* values of the *F_x_*, *F_y_*, *F_z_*, and *T_n_* models are all above 85%, which indicates that the model fits well. The Adeq precision can be used to measure the signal-to-noise ratio, and it can be found that the Adeq precision in the four regression prediction models is greater than 4. This means that the four regression prediction models have sufficient signals. In summary, the established quadratic regression prediction model is valid.

**Figure 4:**
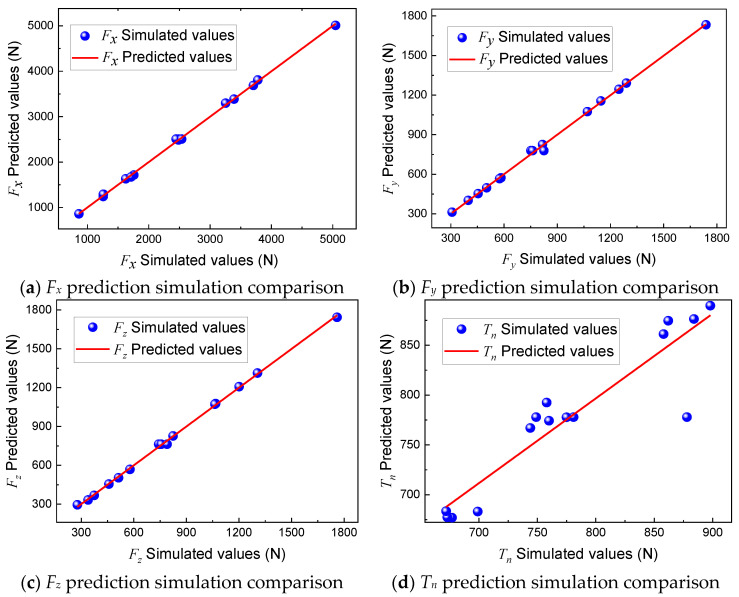
Comparisons of predicted values and simulation values.

Figure 4 shows the relationship between the predicted and simulated test values of *F_x_*, *F_y_*, *F_z_*, and *T_n_*, where the fitted curves are the predicted values and the data points are the simulated values, from which it can be found that the simulated values are almost all on the predicted curve, a small number fluctuate on the predicted curve, and there are no points with large deviations, which proves that the prediction model has good accuracy.

**Figure 5:**
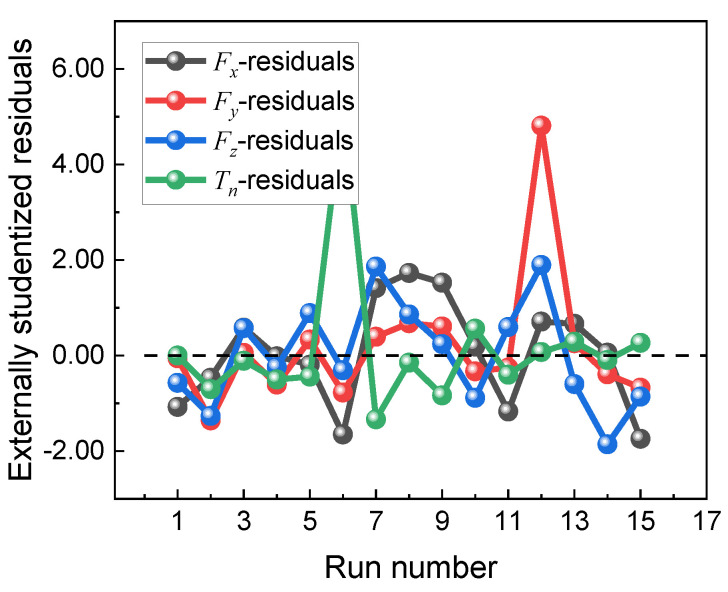
Residual distribution of *F_x_*, *F_y_*, *F_z_*, and *T_n_*.

Figure 5 shows the residual distribution plots of *F_x_*, *F_y_*, *F_z_*, and *T_n_*. It can be observed that the trend of the response values is random, there is no clear quantitative relationship between each residual term, and all residuals fluctuate on the baseline zero line, which indicates that the residual distribution plots of cutting forces in the three directions conform to the law of normal distribution and can measure the relationship between each factor and the response values more accurately.

To further verify the validity of the four regression prediction models (*F_x_*, *F_y_*, *F_z_*, and *T_n_*) established in this paper, it is necessary to conduct simulation tests on these models. Five groups of cutting parameters were randomly selected within the range of cutting parameters selected above for cutting simulation verification, and the specific cutting parameters are shown in Table 6.

The results of the cutting simulation validation are shown in Table 7. It can be found that the predicted values (Pred) of *F_x_*, *F_y_*, *F_z_*, and *T_n_* in five groups of randomly selected cutting parameters are very close to the simulation values (Sim) with a maximum error of 7.5%, which does not exceed 10%, further proving the validity of the four regression prediction models established in this paper.

**Figure 6:**
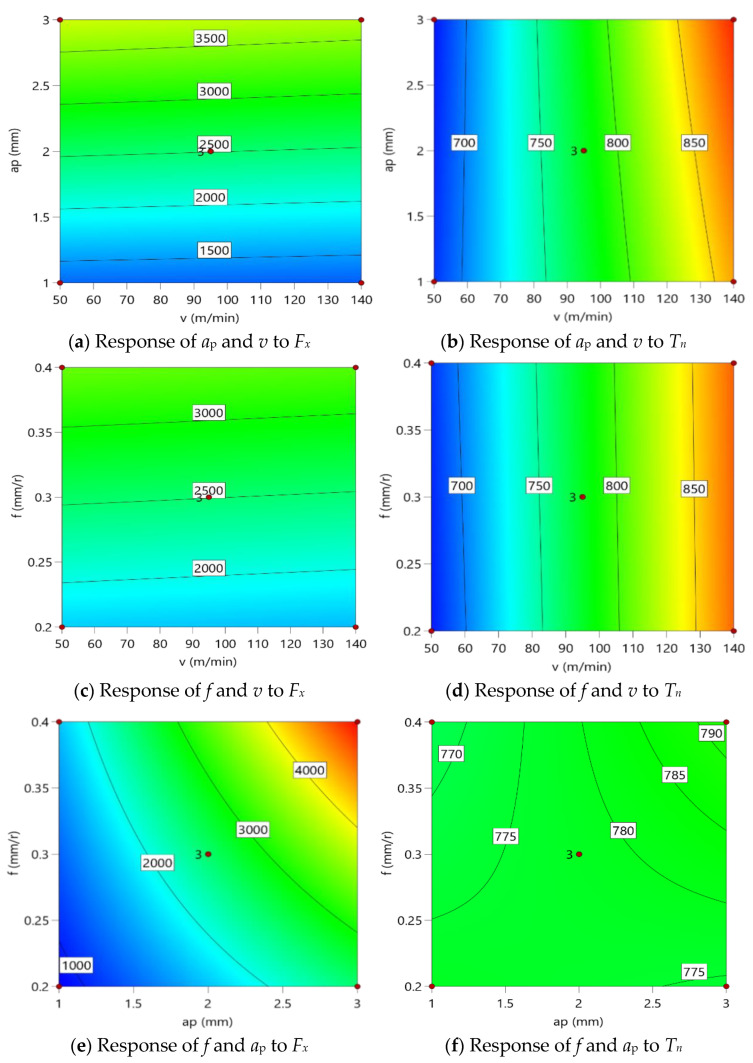
Response surface of *F_x_* and *T_n_*.

Response surface analysis can show the influence law of different factors on the response values. The results of the response surface analysis based on Table 4 are shown in Figure 6, and only the response surfaces of *F_x_* and *T_n_* are shown due to the consistent influence law of the three factors on the three cutting forces.

From Figure 6a,c, it can be found that *F_x_* increases significantly with the increase in *f* and *a_p_*, and the effects of *f* and *a_p_* on *F_x_* are significantly larger than *v*. From Figure 6e, it can be found that the increase in *F_x_* with the increase in *a_p_* is larger than the increase in *F_x_* with the increase in *f*. Therefore, the degrees of effects of *v*, *a_p_*, and *f* on *F_x_* are as follows: *a_p_* > *f* > *v*. The effects of *v*, *a_p_*, and *f* on *F_y_* and *F_z_* are consistent with the degree of influence on *F_x_*. The response surface in Figure 6b,d clearly shows the effects of *v*, *a_p_*, and *f* on *T_n_* with the following degree of influence of the three factors on *T_n_*: *v* > *f* and *v* > *a_p_*. Due to Figure 6f, the effects cannot be analyzed directly; therefore, further analysis of both is required subsequently.

**Figure 7:**
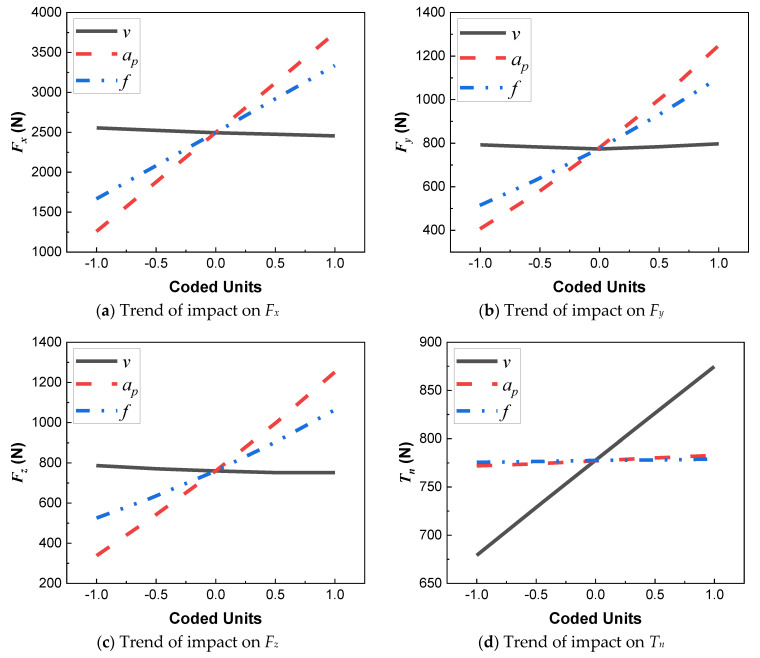
Trends of impact on *F_x_*, *F_y_*, *F_z_*, and *T_n_*.

To further analyze the effects of the three factors *v*, *a_p_*, and *f* on the four response values of *F_x_*, *F_y_*, *F_z_*, and *T_n_*, the single factor influence trend of the four response values was plotted as shown in Figure 7, which mainly encodes the upper and lower limits of each factor as −1 and 1 to achieve the unification of the upper and lower limits of each factor, which can show the effects of different factors on the target response values on the same interval. From Figure 7a–c, it can be seen that the effects of the three factors on *F_x_*, *F_y_*, and *F_z_* are consistent, while the increase in both *f* and *a_p_* increases the cutting force significantly. The larger *f* is, the faster the tool removes the material, and the material to be cut can deform and leave the cutting area in a shorter time, thus creating more resistance to the tool. The larger the *a_p_*, the higher the rate of material removal by the tool per unit time, the larger the tool–chip contact area, and the larger the cutting force. As *v* increases, *F_x_*, *F_y_*, and *F_z_* decrease slightly; this is because the increase in *v* produces a higher cutting temperature when the workpiece material softens in the cutting area, so that the cutting force becomes smaller, but the degree of change is usually small.

From Figure 7d, it can be found that the increase in all three factors increases *T_n_*, among which the change in *v* has the most obvious effect on *T_n_*, which is because as *v* increases, the material removal rate per unit time increases, the power consumption becomes larger, and the cutting heat increases. When *f* and *a_p_* change, the effect on *T_n_* is not obvious, and the effect of *a_p_* is slightly greater. Therefore, the degree of influence of *v*, *a_p_*, and *f* on *T_n_* is as follows: *v* > *a_p_* > *f*.

## 3. Multi-Objective Optimization of Cutting Parameters Based on Genetic Algorithm

After obtaining the cutting force model and the cutting temperature model, to complete the subsequent simulation analysis and machining experiment, based on the genetic algorithm, the cutting force *F_x_* and the material removal rate *Q* were maximized as the optimization objectives, and the cutting speed *v*, the depth of cut *a_p_*, and the feed rate *f* were used as the decision variables to carry out the multi-objective optimization of the cutting parameters within the constraints of the corresponding parameters to obtain a set of optimal cutting parameters.

In multi-objective optimization, the optimization objective needs to be characterized by numerical quantification, and the minimum value of the function of the solved object is the objective. When the maximum objective is solved, the minimum value of its opposite number can be found. The multi-objective optimization model usually consists of multiple decision variables, objective functions, and constraints, and its optimization model can be expressed as follows

(9) $$\left\{\begin{array}{c} minY=F\left(X\right)=\left[F_{1}\left(X\right),F_{2}\left(X\right),\ldots ,F_{n}\left(X\right)\right]\\ A_{i}\left(X\right)\leq 0\;,\;i=1,2,\ldots ,a\\ B_{j}\left(X\right)=0,\;j=1,2,\ldots ,b \end{array}\right.$$

where X($x_{1},x_{2},\ldots ,x_{m}$) is the decision vector; $Y(f_{1},f_{2},\ldots ,f_{n})$ is the target vector.

The multi-objective optimization of cutting force and material removal rate was performed based on a genetic algorithm. To establish a multi-objective optimization model, decision variables, objective functions, and constraints are required. The *v*, *a_p_*, and feed rate *f* are used as decision variables, so the decision variable X can be expressed as

(10) $$X={\left(v,a_{p},f\right)}^{T}$$

In the cutting process, the values of *F_y_* and *F_z_* are small compared to the main cutting force *F_x_*. To avoid complicating the optimization problem, the optimization objectives need to be reduced, and since the growth law of *F_x_*, *F_y_*, and *F_z_* is the same, only the main cutting force *F_x_* was considered for optimization. At the same time, the material removal rate *Q* was also taken as the optimization objective to take into account the machining efficiency of the workpiece. Specifically, with the minimum cutting force *F_x_* and the maximum material removal rate *Q* as the optimization objectives, it can be found that there is a conflict between the two in the selection of cutting dosage, so a multi-objective optimization model can be established for both, where the minimum main cutting force model can be established according to Equation (5):

(11) $$F_{1}\left(X\right)=\min F_{x}$$

According to the process characteristics of the boring process, the material removal rate *Q* can be expressed as

(12) $$Q=1000va_{p}f$$

As a result, the maximum material removal rate model can be expressed as

(13) $$F_{2}\left(X\right)=\min\left(-Q\right)$$

In terms of constraints, the decision variables X are mainly constrained. The ranges of *v* and *f* are consistent with the above, and their constraint ranges are 50~140 m/min and 0.2~0.4 mm/rev, respectively. The *a_p_* has a large impact on cutting, and its constraint range is set to 0.5~1.3 mm to avoid the occurrence of chatter.

Through the above decision variables, objective functions and constraints, a multi-objective optimization model is established. In the parameter setting of the genetic algorithm, the initial population size is set to 100, which is randomly generated by the corresponding function; The selection strategy is the tournament function, whose size is 2. The crossover probability is set to 0.8. In terms of variation probability, the adaptive feasible variation function is used to obtain better optimization results. The function tolerance is 1 × 10^−6^, and the population proportion of the Pareto front is 0.35.

**Figure 8:**
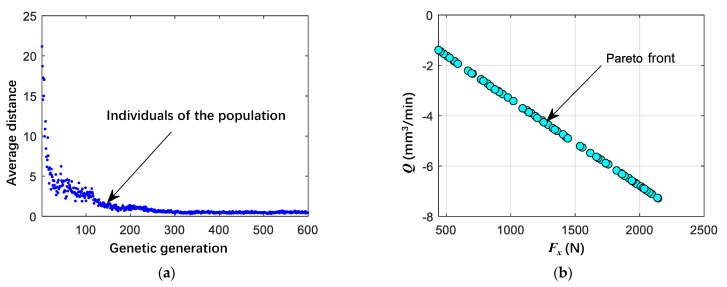
Multi-objective optimization results for cutting force *F_x_* and material removal rate *Q*. (**a**) The average distance between individuals and the population; (**b**) Pareto front.

Based on MATLAB, the results are shown in Figure 8, and a Pareto optimal solution set was obtained, as shown in Table 8. Figure 8a shows the average distance between individuals of the population in each generation, and it can be found that when the population is randomly generated in the initial few generations, the arrangement between the populations is relatively discrete; thus, the average distance is larger, and the value of fitness gradually becomes larger as the number of genetic generations increases. When the evolution reaches 300 generations, the fitness no longer increases, and, at this time, the individuals of the population approximately overlap with each other, and the distance between them tends to 0, indicating that the optimal solution set has been solved at this time. Figure 8b shows the Pareto front, which consists of the optimal solution set and is not a discrete distribution but approximately a straight line, indicating a good solution effect.

Considering the magnitude of cutting force *F_x_* and material removal rate *Q*, the sixth group of cutting parameters in Table 8 were selected as the optimal cutting parameters, which was used for the subsequent machining experiment.

## 4. TC4 Tube Deep Hole Boring Experiment

### 4.1. Experimental Conditions

**Figure 9:**
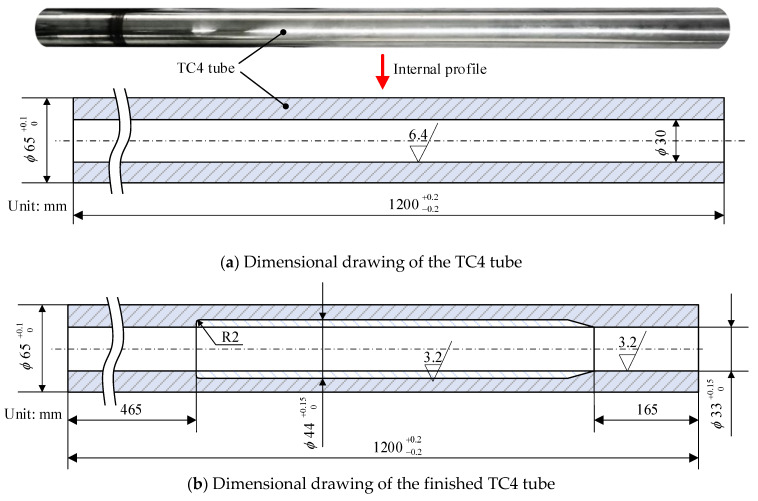
TC4 tube.

The object machined in this experiment is a deep hole drilled TC4 tube with an external dimension of *ϕ*65 mm × 1200 mm, an inner hole dimension of *ϕ*30 mm, and an L/D ratio of 40, as shown in Figure 9a. It is required to use the deep bottle hole tool to machine the deep hole tube, and the specific machining requirements are as follows: the external dimensions remain unchanged, the basic size of the inner hole is expanded to *ϕ*33 mm, the expanded size of the middle part is *ϕ*44 mm, and the L/D ratio is 36.36, as shown in Figure 9b. The final roughness of the deep hole wall must reach Ra 3.2 µm, and the straightness must reach 0.5 mm/1000 mm.

**Figure 10:**
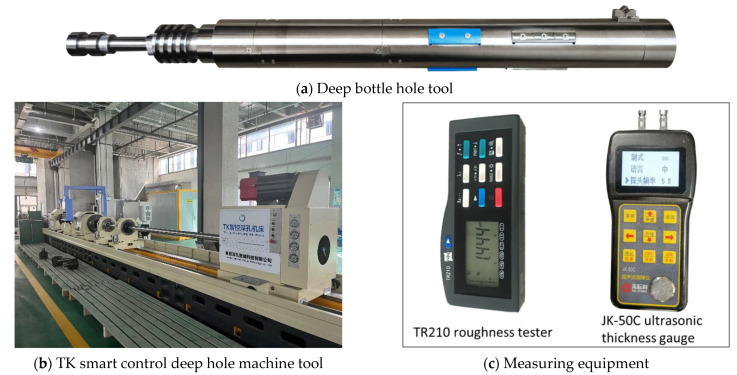
Experimental equipment.

The deep hole boring experiment was implemented based on the special deep bottle hole tool and TK smart control deep hole machine tool, as shown in Figure 10a,b. The experimental equipment and measuring equipment involved in this experiment are the TR210 roughness tester and the JK-50C ultrasonic thickness gauge, which are used to measure the roughness of the hole and the thickness of the tube wall, respectively, and the equipment is shown in Figure 10c.

To facilitate chip removal and reduce the cutting temperature, 69-1 emulsion was selected as the cutting fluid, and the flow rate and pressure of the cutting fluid were 150 L/min and 2 MPa, respectively. The insert parameters selected for this machining experiment were consistent with the cutting simulation.

### 4.2. Machining Process

Due to the complex internal profile of the deep bottle hole and the relatively large length and diameter, it is impossible to realize the one-time formation of the inner hole. This is mainly because as the length of the deep bottle hole tool increases, its overall stiffness must decrease, and when machining, there is a large vibration, which not only causes damage to the tool but also produces a larger roughness, higher residual stress, and lower dimensional accuracy on the inner hole surface of the deep bottle holes. Therefore, considering the machining characteristics of deep bottle holes and the push boring method, which is prone to causing the destabilization of the boring bar, a sectional boring process is used. This process divides the entire inner hole of the deep bottle hole into multiple segments according to a certain distance or different inner hole profiles.

The cutting parameters used in the sectional boring process are the optimal cutting parameters (*v*, *a_p_*, *f*) = (50 m/min, 1 mm, 0.26 mm/rev) obtained above. The lengths of the three parts of TC4 tubing are 165 mm, 570 mm, and 465 mm from left to right, of which the lengths of the three sections of the middle part are 30 mm, 270 mm, and 270 mm.

**Figure 11:**
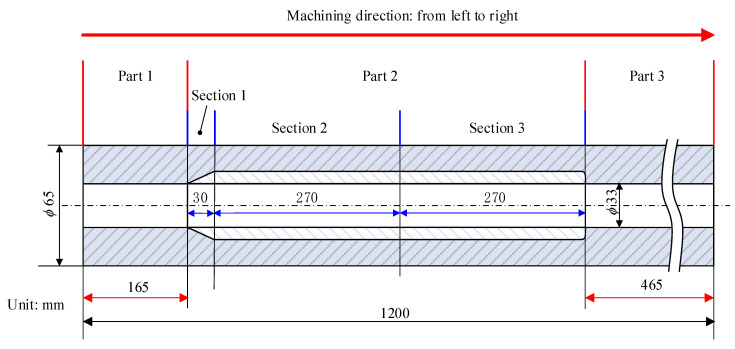
Sectional boring process TC4 tube.

During the machining of the TC4 tube, the tool is extended into the hole from the right end of the workpiece in Figure 11, and the machining starts from the left to the right in a sectional boring process. The cutting fluid is supplied by the cutting fluid supply apparatus and flows into the cutting area from the annular space formed by the inner wall of the right end of the workpiece and the outer wall of the deep bottle hole tool, which in turn carries away the cutting heat and chips from the cutting area and is discharged from the left port of the workpiece.

### 4.3. Analysis of Experimental Results

**Figure 12:**
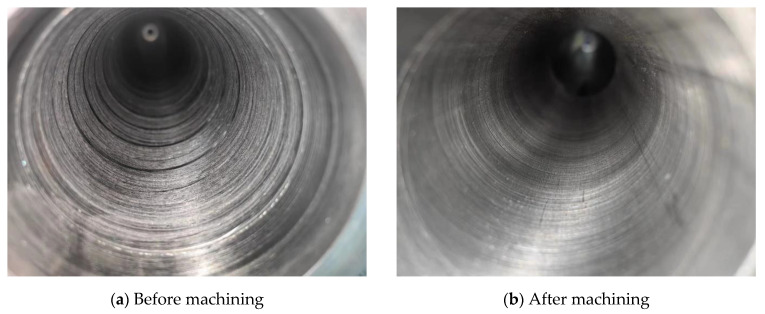
Deep hole boring result.

The machined, finished workpiece is shown in Figure 12. The roughness at the left end where the cutting quality of the TC4 tube is poor was measured using a TR210 roughness tester. After the measurement, the surface roughness of the hole before machining was Ra 6.3 µm, and the surface roughness of the hole after machining reached Ra 3.2 µm, which proves that the quality of the inner hole of the TC4 tube was improved after machining.

**Figure 13:**
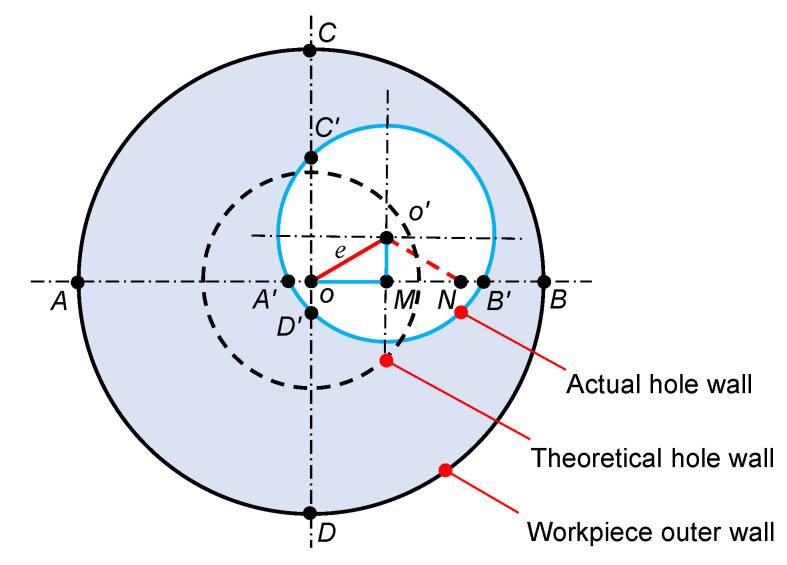
Axial eccentric hole profile.

Using the JK-50C ultrasonic thickness gauge to measure the wall thickness of the TC4 tube, the straightness is measured by measuring the eccentricity (*e*) of the hole axis at 1000 mm hole depth, starting from the right end of the TC4 tube in Figure 11. The *e* is obtained from the wall thickness value of any four equal nodes on the circumference of the requested section and calculated by Equation (14), and the cross-sectional view of the axial eccentric hole is shown in Figure 13. The four wall thicknesses $AA^{\prime }$, $BB^{\prime }$, ${CC}^{\prime }$, and ${DD}^{\prime }$ measured by the ultrasonic thickness gauge are 10.94 mm, 10.03 mm, 10.71 mm, and 10.33 mm, respectively. The *e* is 0.493 mm after calculation by Equation (14), and its straightness is 0.493 mm/1000 mm, which meets the requirement of straightness.

(14) $$e=\sqrt{{\left(\frac{AA^{\prime }-BB^{\prime }}{2}\right)}^{2}+{\left(\frac{{DD}^{\prime }-{CC}^{\prime }}{2}\right)}^{2}}$$

**Figure 14:**
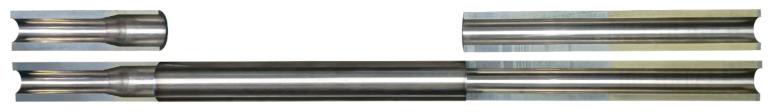
Finished TC4 tube.

After wire cutting and grinding the finished TC4 tube section as shown in Figure 14, it can be found that on the left side of the workpiece at the transition, the roughness of its wall is high, resulting in very slight tool marks, which is due to the deep bottle hole tool in the machining here; the tool head part of the insert needs to withstand a large cutting force to retract out, resulting in a slight vibration of the tool under very high external excitation conditions, resulting in a trace of tool marks, but it still meets the machining quality requirements. A deep bottle hole with a depth of cut of 5.5 mm and an L/D ratio of 36.36 is machined, and the finished TC4 tube can be used in parts such as aircraft landing gear and low-pressure turbine shafts for gas turbines.

## 5. Conclusions

In this study, finite element cutting simulations and a deep hole boring experiment were conducted mainly for the machining of deep bottle holes, and the main conclusions obtained are as follows.

(1) The cutting forces *F_x_*, *F_y_*, and *F_z_* and the cutting temperature *T_n_* with 15 sets of cutting parameters were output based on the response surface method and through the finite element software, and the regression prediction model of four response values was established with this. The degree of influence of cutting speed *v*, depth of cut *a_p_*, and feed rate *f* on *F_x_*, *F_y_*, and *F_z_* was analyzed: *a_p_* > *f* > *v*; the degree of influence on *T_n_*: *v* > *a*_p_ > *f*.

(2) Based on the multi-objective optimization of cutting force *F_x_* and material removal rate *Q* by the genetic algorithm, a set of optimal cutting parameters were obtained, namely (*v*, *a_p_*, *f*) = (139.41 m/min, 1.12 mm, 0.27 mm/rev).

(3) Based on the optimal cutting parameters, a TC4 tube with a complex profile with an L/D ratio of 36.36 was machined with a hole surface roughness of Ra 3.2 µm and a straightness of 0.5 mm/1000 mm, and the finished TC4 tube machined can be fabricated into deep bottle-hole parts such as aircraft landing gear.

## Figures and Tables

**Table 1:** Cutting parameters and levels.

Cutting Parameters	Level		
	−1	0	1
*v* (m/min)	50	95	140
*a_p_* (mm)	1	2	3
*f* (mm/rev)	0.2	0.3	0.4

**Table 2:** Material parameters of TC4 and YG8 cemented carbide.

Materials	Density (g/cm^3^)	Elasticity Modulus (MPa)	Poisson’s Ratio	Thermal Conductivity (W/m·K)	Expansion Coefficient (10^6^·m/K)	Specific Heat (J/Kg·K)
TC4	4.43	113,000	0.34	7.955	9.1	546
YG8	14.6	600,000	0.22	75.4	4.5	220

**Table 3:** Johnson–Cook plasticity and damage parameters for TC4 [17].

*A* (MPa)	*B* (MPa)	*C*	*n*	*m*	*T_m_* (°C)	*T_r_* (°C)	*D*_1_	*D*_2_	*D*_3_	*D*_4_	*D*_5_
783	498	0.028	0.28	1	1680	20	−0.09	0.25	−0.5	0.014	3.87

**Table 4:** Results of cutting simulation.

Number	*v* (m/min)	*a_p_* (mm)	*f* (mm/rev)	*F_x_* (N)	*F_y_* (N)	*F_z_* (N)	*T_n_* (°C)
1	50	3	0.3	3779	1290	1306	677
2	95	2	0.3	2487	751	742	749
3	140	1	0.3	1258	454	374	858
4	95	3	0.2	2485	817	824	760
5	140	2	0.2	1625	502	513	862
6	95	2	0.3	2446	761	757	878
7	95	3	0.4	5047	1739	1760	758
8	50	2	0.2	1758	583	578	673
9	95	1	0.4	1712	575	458	744
10	50	2	0.4	3391	1070	1067	699
11	50	1	0.3	1260	398	339	672
12	95	2	0.3	2537	825	790	781
13	140	3	0.3	3706	1248	1201	898
14	95	1	0.2	862	307	278	775
15	140	2	0.4	3254	1146	1063	884

**Table 5:** Results of the ANOVA for model.

Model	Sum of Square	Degrees of Freedom	Mean Square	*F-*Value	*p-*Value	Lack of Fit	*R* ^2^	Adeq Precision
*F_x_*	1.863 × 10^7^	6	3.105 × 10^6^	1740.25	<0.0001	0.8120	0.9992	143.8119
*F_y_*	2.213 × 10^6^	9	2.459 × 10^5^	330.25	<0.0001	0.1030	0.9983	63.7518
*F_z_*	2.397 × 10^6^	9	2.664 × 10^5^	576.14	<0.0001	0.6112	0.9990	82.5474
*T_n_*	7.704 × 10^4^	6	1.284 × 10^4^	7.61	0.0058	0.9636	0.8509	7.5819

**Table 6:** Verification set of cutting parameters.

Number	*v* (m/min)	*a_p_* (mm)	*f* (mm/rev)
1	50	1	0.26
2	64	1.2	0.31
3	125	1.5	0.22
4	116	2.1	0.28
5	83	1.6	0.35

**Table 7:** Cutting simulation verification results.

Number	*F_x_* (N)			*F_y_* (N)			*F_z_* (N)			*T_n_* (°C)		
	Pred	Sim	Error	Pred	Sim	Error	Pred	Sim	Error	Pred	Sim	Error
1	1129	1090	3.6%	371	383	3.1%	311	333	6.6%	685	712	3.8%
2	1584	1568	1.0%	482	462	4.3%	427	410	4.1%	711	680	4.6%
3	1369	1376	0.5%	423	430	1.6%	417	405	3.0%	839	861	2.6%
4	2436	2351	3.6%	756	727	4.0%	740	738	0.3%	823	860	4.3%
5	2353	2388	1.5%	729	680	7.5%	689	659	4.6%	750	803	6.6%

**Table 8:** Pareto optimal solution set (partial).

Number	*v* (m/min)	*a*_p_ (mm)	*f* (mm/rev)	*F_x_* (N)	Q (mm^3^/min)
1	139.47	1.21	0.38	1896	−64,128
2	139.59	1.08	0.35	1560	−52,765
3	139.36	1.22	0.34	1712	−57,807
4	139.87	1.29	0.39	2074	−70,369
5	139.49	1.18	0.38	1849	−62,547
6	139.41	1.12	0.27	1252	−42,158
7	139.26	0.79	0.37	1206	−40,706
8	139.32	0.68	0.27	771	−25,579
9	139.83	1.23	0.37	1876	−63,637
10	139.83	0.89	0.39	1430	−48,535

## Data Availability

Not applicable.
